# Gene co-expression networks shed light into diseases of brain iron accumulation

**DOI:** 10.1016/j.nbd.2015.12.004

**Published:** 2016-03

**Authors:** Conceição Bettencourt, Paola Forabosco, Sarah Wiethoff, Moones Heidari, Daniel M. Johnstone, Juan A. Botía, Joanna F. Collingwood, John Hardy, for the UK Brain Expression Consortium (UKBEC), Elizabeth A. Milward, Mina Ryten, Henry Houlden

**Affiliations:** a Department of Molecular Neuroscience, UCL Institute of Neurology, London, UK; b Istituto di Ricerca Genetica e Biomedica CNR, Cagliari, Italy; c School of Biomedical Sciences and Pharmacy, The University of Newcastle, Callaghan, NSW, Australia; d Centre for Bioinformatics, Biomarker Discovery and Information-Based Medicine, The University of Newcastle, Callaghan, NSW, Australia; e Bosch Institute and Discipline of Physiology, University of Sydney, NSW, Australia;; f Department of Medical and Molecular Genetics, King's College London, London, UK; g School of Engineering, University of Warwick, Coventry, UK; h Department of Clinical and Experimental Epilepsy, UCL Institute of Neurology, London, UK; i Center for Neurology and Hertie Institute for Clinical Brain Research, Eberhard-Karls-University, Tübingen, Germany

**Keywords:** Human brain, Whole-transcriptome analysis, WGCNA, NBIA, Iron metabolism

## Abstract

Aberrant brain iron deposition is observed in both common and rare neurodegenerative disorders, including those categorized as Neurodegeneration with Brain Iron Accumulation (NBIA), which are characterized by focal iron accumulation in the basal ganglia. Two NBIA genes are directly involved in iron metabolism, but whether other NBIA-related genes also regulate iron homeostasis in the human brain, and whether aberrant iron deposition contributes to neurodegenerative processes remains largely unknown. This study aims to expand our understanding of these iron overload diseases and identify relationships between known NBIA genes and their main interacting partners by using a systems biology approach.

We used whole-transcriptome gene expression data from human brain samples originating from 101 neuropathologically normal individuals (10 brain regions) to generate weighted gene co-expression networks and cluster the 10 known NBIA genes in an unsupervised manner. We investigated NBIA-enriched networks for relevant cell types and pathways, and whether they are disrupted by iron loading in NBIA diseased tissue and in an in vivo mouse model.

We identified two basal ganglia gene co-expression modules significantly enriched for NBIA genes, which resemble neuronal and oligodendrocytic signatures. These NBIA gene networks are enriched for iron-related genes, and implicate synapse and lipid metabolism related pathways. Our data also indicates that these networks are disrupted by excessive brain iron loading.

We identified multiple cell types in the origin of NBIA disorders. We also found unforeseen links between NBIA networks and iron-related processes, and demonstrate convergent pathways connecting NBIAs and phenotypically overlapping diseases. Our results are of further relevance for these diseases by providing candidates for new causative genes and possible points for therapeutic intervention.

## Highlights

• Weighted gene co-expression analysis gives insights on brain disease biology.
• Excessive brain iron overload disrupts normal basal ganglia gene networks.
• Basal ganglia iron accumulation may determine disease progression.

## 1 Introduction

Aberrant brain iron deposition occurs in common neurodegenerative disorders (e.g. Parkinson's and Alzheimer's diseases (Oakley et al., 2007, Tao et al., 2014)), and more prominently in rare inherited diseases categorized as Neurodegeneration with Brain Iron Accumulation (NBIA) (Dusek et al., 2012). Iron is essential for normal brain function and is heterogeneously and dynamically distributed in the brain (Piñero and Connor, 2000, Rouault, 2013). The basal ganglia are among the regions with highest iron levels, and the highest concentrations are observed in oligodendrocytes. Our understanding of brain iron metabolism and how it relates to neurodegeneration and disease is limited due to the inability to distinguish brain cell types via non-invasive techniques (e.g. MRI) and poor understanding of how iron traffics in the brain to adequately supply neurons, astrocytes, oligodendrocytes and microglia (Rouault, 2013, Kruer et al., 2012, Schneider et al., 2013, Levi and Finazzi, 2014).

NBIA disorders are clinically characterized by a progressive movement disorder with complicating symptoms that can vary significantly in terms of range and severity, and frequently include neuropsychiatric disturbances, such as cognitive deficits, personality changes with impulsivity and violent outbursts, depression, emotional lability, and obsessive compulsive disorder (Gregory et al., 2009). This clinically heterogeneous picture is unified by focal brain iron accumulation, predominantly in the basal ganglia (Kruer et al., 2012, Schneider et al., 2013, Levi and Finazzi, 2014). Ten NBIA genetic diseases have already been defined (Table 1), yet many cases remain genetically undiagnosed (Levi and Finazzi, 2014). Two NBIA genes (*FTL* and *CP*) are directly involved in iron metabolism, but it remains elusive whether other NBIA genes also regulate iron-related processes in the human brain.

We analyzed whole-transcriptome gene expression data from normal human brain and used weighted gene co-expression network analysis (WGCNA) to group NBIA genes into modules in an unsupervised manner (Langfelder and Horvath, 2008, Oldham et al., 2006, Oldham et al., 2008, Zhang and Horvath, 2005). This systems-biology approach (Lee et al., 2004, Stuart et al., 2003) enables the identification of modules of biologically related genes that are co-expressed and co-regulated (Oldham et al., 2008, Konopka, 2011, Rosen et al., 2011, Winden et al., 2009), and can give insights on cell-specific molecular signatures (Oldham et al., 2008, Bettencourt et al., 2014, Forabosco et al., 2013). The main goal of this study was to expand our understanding of these iron overload diseases by identifying relationships and shared molecular pathways between known NBIA genes, and unraveling transcriptionally linked novel candidates to facilitate discovery of new genes associated with these diseases and of possible entry points to therapeutic intervention.

## 2 Subjects and methods

### 2.1 Human control brain samples and whole-genome expression profiling

Brain samples from 101 adult individuals were collected by the Medical Research Council (MRC) Sudden Death Brain and Tissue Bank (Millar et al., 2007). All brains samples were neuropathologically normal, had fully informed consent and were authorized for ethically approved scientific investigation (Research Ethics Committee 10/H0716/3). Within the frame of the UK Brain Expression Consortium (UKBEC), total RNA was isolated and processed for analysis using Affymetrix Exon 1.0 ST Arrays (Affymetrix UK Ltd., High Wycombe, UK) as described elsewhere (Bettencourt et al., 2014, Forabosco et al., 2013, Trabzuni et al., 2011).

### 2.2 Weighted gene co-expression network analysis in the adult normal human brain

Using whole-transcriptome gene expression data, NBIA genes/transcripts were assigned to co-expression modules (arbitrary colors) identified through WGCNA (Langfelder and Horvath, 2008, Zhang and Horvath, 2005). For the adult brain network analysis (10 brain regions from 101 adult individuals, UKBEC data) a total of 15,409 transcripts (13,706 genes) passing quality control were used to identify modules, and 3743 additional transcripts (3541 genes) were assigned to modules based on their highest module membership, as previously described (Forabosco et al., 2013). Briefly, the WGCNA network was constructed for each tissue using a signed network with power (Beta) of 12 to achieve a scale-free topology. A dissimilarity matrix based on topological overlap measure (TOM) was used to identify gene modules (i.e. densely interconnected and co-expressed genes), through a dynamic tree-cutting algorithm. More details are given by Forabosco et al. (2013).

Module preservation statistics (Z summary) were calculated as previously described (Langfelder et al., 2011) to assess how well modules from one tissue are preserved in another tissue. Based on the empirical thresholds proposed by Langfelder et al. (Langfelder et al., 2011), Z summary scores above 10 indicate strong evidence for module preservation across brain regions. To determine the relevance of each gene in a module, we estimated the module membership (MM), also known as eigengene-based connectivity. Gene interconnections within NBIA transcript-enriched modules were further investigated using VisANT (http://visant.bu.edu) (Hu et al., 2004).

Hypergeometric distribution was used to evaluate the overrepresentation of NBIA and iron-related gene transcripts in the gene co-expression modules (nominal p-values < 0.05 were considered significant). To further assess the statistical significance of the enrichment of NBIA genes in given putamen modules, we developed a permutation test to estimate the probability that *g* genes will be found together by chance within a module of size equal or less than *m* for a given partition of genes *G* = {*g*_1_,…, *g*_n_}, arranged into *k* modules *P* = {*p*_1_,…,*p*_k_}, such that each gene *g*_i_ belongs only to a single module. To estimate the probability of finding *g* genes in a module of size *m* or less in partition *P*, we randomly permuted the genes in *G* in a list and annotated each gene in that list with the module in *P* to which the gene belongs. Then we repeated the following procedure 10^6^ times, randomly choosing *g* positions from the list and checking whether the corresponding genes were annotated with the same module and the module had size *m* or less. Finally, the probability of finding by chance *g* genes in a module of size *m* or less was estimated by dividing by 10^6^ the number of times *g* genes were found together in such modules.

### 2.3 Validation of basal ganglia co-expression networks in independent data sets

We used independent and publicly available basal ganglia gene expression networks (Oldham et al., 2008), from 27 adult caudate nucleus samples, to investigate whether our NBIA-containing modules overlap with modules in those previously published networks. We also used the only publicly available basal ganglia pediatric whole-transcriptome gene expression data set (Kang et al., 2011) (7 striatum samples from clinically unremarkable donors with ages ranging from 2 to 19 years) to perform WGCNA (Langfelder and Horvath, 2008, Zhang and Horvath, 2005). We generated pediatric signed networks using a power (Beta) of 33 and a height of 0.2. A total of 15,285 genes passing quality control were used to identify modules. Fisher's exact test was used to determine the significance of the overlap between distinct networks (nominal p < 0.05 was considered significant).

### 2.4 Gene expression analysis in NBIA diseased basal ganglia tissue

Additional validation studies investigated whether the NBIA-containing modules overlap with differentially expressed genes in human NBIA disorders. We used post-mortem basal ganglia tissue from two adults, one male and one female (66 and 81 years at death, respectively), with a confirmed clinicopathological diagnosis of NBIA (Canadian Brain Tissue Bank, University of Toronto, Canada), and two age- and gender-matched adults with no diagnosed neurological conditions (Newcastle Brain Tissue Resource, University of Newcastle, UK). All brain tissue was obtained with fully informed consent and the study was approved by the Human Research Ethics Committee of the University of Newcastle, Australia (H-2010-1219). Total RNA was obtained as previously described (Johnstone et al., 2012, Acikyol et al., 2013), and arrays performed using the Illumina HumanHT-12 v4 Expression BeadChip (Illumina, San Diego, USA). Following Cubic Spline normalization in GenomeStudio Gene Expression Module (Illumina, v2010.3), genes were considered differentially expressed if the fold-change of the mean NBIA signal relative to the mean control signal for each brain region was at least 1.5. The small sample size prevented statistical comparison of means and further analysis. Chi-square testing determined the significance (nominal p < 0.05) of the overlap between differentially expressed genes in NBIA brain and NBIA-enriched co-expression modules in normal human brain.

### 2.5 Gene expression analysis in *Hfe*^*−*/*−*^* xTfr2*^*mut*^*AKR* mice brains

The *Hfe*^*−*/*−*^ x*Tfr2*^mut^ mouse model of iron overload was generated previously (Delima et al., 2012) by crossing mice with deletion of the *Hfe* gene (Hfe^−/−^) (Johnstone et al., 2012, Zhou et al., 1998, Fleming et al., 2001, Trinder et al., 2002) with mice harboring the p.Y245X nonsense mutation in the transferrin receptor 2 gene (*Tfr2*^*mut*^) (Acikyol et al., 2013, Fleming et al., 2002, Drake et al., 2007, Chua et al., 2010). Mice were on an AKR genetic background, which manifests a strong iron loading phenotype (Fleming et al., 2001, McLachlan et al., 2011). Further details of the *Hfe*^*−*/*−*^ x*Tfr2*^mut^ model are provided elsewhere (Delima et al., 2012).

All protocols were approved by the Animal Ethics Committees of the University of Western Australia. Male wild-type and *Hfe*^*−*/*−*^ x*Tfr2*^mut^ mice were fed standard mouse chow from weaning. At 10 weeks of age, *Hfe*^*−*/*−*^ x*Tfr2*^mut^ mice were switched to an iron-supplemented diet containing 2% carbonyl iron (Sigma Aldrich, St. Louis, MO, USA) for 3 weeks. At 13 weeks of age, mice were anesthetized (50 mg/kg ketamine, 10 mg/kg xylazine; Troy Laboratories Pty Ltd., Smithfield, NSW, Australia) and perfused transcardially with isotonic saline. Brain tissue was collected and snap frozen in liquid nitrogen.

RNA isolation and microarray analysis were performed as described in the previous section except that samples (n = 4 per group) were hybridized to Illumina Sentrix MouseRef-8 v2.0 BeadChip microarrays, as previously described (Johnstone et al., 2012, Acikyol et al., 2013). Microarray data were subjected to Average or Cubic Spline normalization in GenomeStudio Gene Expression Module (Illumina, v2010.3) and differential expression determined using either GenomeStudio (error model Illumina Custom) or GeneSpring GX 7.3 (Agilent Technologies, Santa Clara, CA, USA) as described elsewhere (Johnstone et al., 2012), generating four lists of differentially-expressed genes. Analyses considered both the union of these four lists (i.e. genes calculated as differentially expressed by at least one of the above mentioned methods) to minimize false negatives, and the intersection (i.e. genes calculated as differentially expressed by all four of the above mentioned methods) to minimize false positives, as detailed elsewhere (Johnstone et al., 2013). A subset of differentially expressed genes was selected for further investigation in additional biological replicates (n = 7 per group) using real-time RT-PCR (further details are available upon request).

### 2.6 Functional annotations and enrichment analysis

To evaluate the biological and functional relevance of NBIA gene expression networks g:Profiler (http://biit.cs.ut.ee/gprofiler/) (Reimand et al., 2007, Reimand et al., 2011) was used. Only genes present in the adult brain networks were used as background for this analysis. Overrepresentation of Gene Ontology (GO) categories, KEGG pathways, and Human Phenotype Ontology (HPO) terms was examined. The g:SCS algorithm was used to account for multiple testing correction, and corrected p < 0.05 was considered significant.

## 3 Results

### 3.1 Regional expression of NBIA genes correlates with the likelihood of white matter involvement

**Fig. 1:**
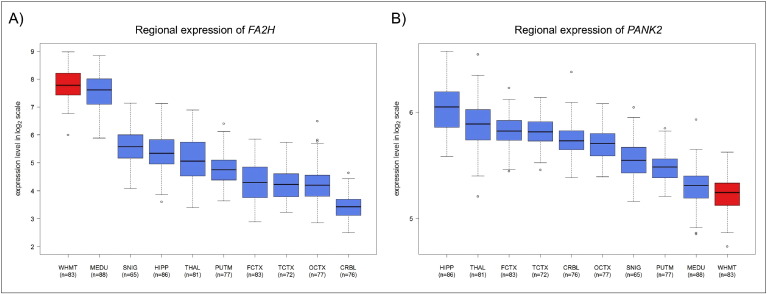
Expression patterns of NBIA genes in pathologically confirmed normal human brains. The following brain regions were studied: cerebellum (CRBL), frontal cortex (FCTX), hippocampus (HIPP), medulla (MEDU), occipital cortex (OCTX), putamen (PUTM), substantia nigra (SNIG), temporal cortex (TCTX), thalamus (THAL), and white matter (WHMT, highlighted in red). A) Example of brain expression patterns of an NBIA gene (*FA2H*) which presents the highest expression levels in the white matter. B) Example of brain expression patterns of an NBIA gene (*PANK2*) which presents the lowest expression levels in the white matter.

Our data and data from the Human Brain Transcriptome project (http://hbatlas.org) (Kang et al., 2011, Johnson et al., 2009) show that NBIA genes are highly expressed in the human brain throughout development and aging. Analysis of 10 brain regions demonstrates that the basal ganglia (putamen and substantia nigra herein studied) are usually not among the regions with the highest expression levels of NBIA genes (Fig. 1). Strikingly, NBIA genes typically associated with white matter changes (Table 1) exhibit the highest expression levels in the white matter (*FTL*, *FA2H*, *DCAF17*, *CP*, and *PLA2G6*), while those not associated with white matter involvement (e.g. *PANK2*) exhibit their lowest levels in this region (Fig. 1), suggesting a relationship between the pattern of gene expression and the observed pathology.

### 3.2 Gene co-expression network analysis identifies basal ganglia NBIA-enriched modules

**Fig. 2:**
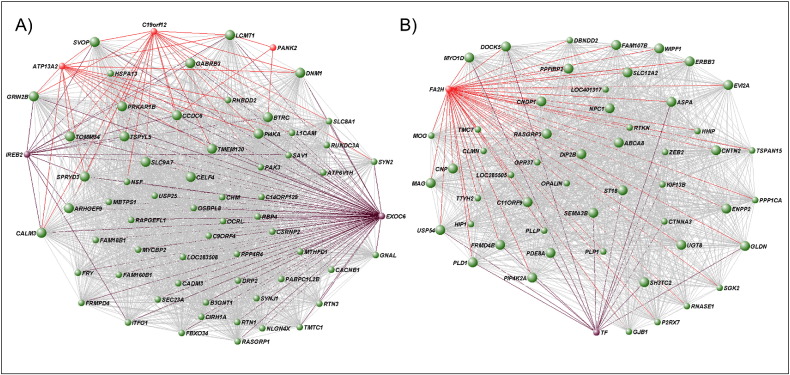
Network representation of the putamen NBIA-enriched modules generated using VisANT (http://visant.bu.edu) (Hu et al., 2004). A) Brown module, showing genes connected with a topological overlap measure (TOM) > 0.08. B) Green module, showing genes connected with a TOM > 0.18. NBIA genes are highlighted in red and iron-related genes are in purple. Given the TOM cut-offs used, not all NBIA and iron-related genes could be included. The biggest circles represent the top hub genes in each module, which stands for the most interconnected genes in the module. Note the connections of NBIA genes and iron-related genes with the same hub genes.

To better understand the functional relationship between NBIA genes, we applied WGCNA to whole-transcriptome data, and focused on basal ganglia modules (Table 2). The substantia nigra shows no significant clustering of NBIA genes. The putamen, however, shows the highest clustering in all 10 brain regions analyzed, consistent with clinicopathological features found in NBIAs converging on basal ganglia involvement. Only 4/20 putamen gene co-expression modules contain NBIA transcripts (Table 2), with a statistically significant clustering in the brown (*PANK2*, *ATP13A2*, *C19orf12*, and *COASY*; hypergeometric distribution p = 0.003; permutation test p = 1.99 × 10^− 4^) and green (*FTL*, *DCAF17*, and *FA2H*; hypergeometric distribution p = 0.021; permutation test p = 2.19 × 10^− 4^) modules. Fig. 2 shows interconnections of genes within these NBIA-enriched modules. All putamen NBIA-containing modules are well preserved across all 10 brain regions (Z summary scores > 10) (Langfelder et al., 2011), which is in line with the clinicopathological complexity of NBIA disorders involving other brain regions (Schneider et al., 2013, Levi and Finazzi, 2014, Hogarth, 2015, Kruer, 2013).

The putamen along with the caudate nucleus forms the striatum, the primary recipient of inputs to the basal ganglia system (Delgado, 2007). To validate our networks, we determined whether our NBIA-containing modules are composed of the same genes as those in publicly available caudate nucleus networks (Oldham et al., 2008) and identified a highly significant overlap between these two basal ganglia networks (e.g. our brown module vs their M16C module: p = 1.38 × 10^− 22^; our green module vs their M9C module: p = 2.69 × 10^− 166^). Furthermore, as NBIA disorders often have a childhood onset, we constructed pediatric gene co-expression networks. The greatest overlap between the adult NBIA-enriched brown and green modules, and the pediatric striatum modules occurs with the striatum aliceblue (p = 2.69 × 10^− 45^) and lightyellow (p = 4.52 × 10^− 80^) modules, respectively. Five out of 10 NBIA genes cluster in the aliceblue module (p = 0.04), 3 of which (*ATP13A2*, *COASY*, and *PANK2*) belong to the adult brown module.

### 3.3 Glial as well as neuronal cell types are implicated in basal ganglia NBIA networks

We next asked whether the putamen NBIA networks can provide information on specific cell types that may be involved in the origin of NBIA disorders. A statistically significant enrichment for neuronal markers (Lein et al., 2007, Cahoy et al., 2008), including *GRIN2B* (99th quantile) and *SYT1* (83rd quantile), was found in the putamen brown module (Table 3). *GAD2* gene (83rd quantile), a marker for GABAergic neurons (Kodama et al., 2012), is also present. Conversely, an overrepresentation of oligodendrocyte markers (Lein et al., 2007, Cahoy et al., 2008), including *MAG* (98th quantile), *MOG* (96th quantile), and *OLIG2* (57th quantile), was found in the putamen green module. This module contains the NBIA gene *FA2H* (84th quantile), also previously described as an oligodendrocyte-enriched gene (Lein et al., 2007, Cahoy et al., 2008). The other two NBIA-containing modules, including the one with *CP*, resemble astrocytic signatures. Overlapping modules from the caudate nucleus networks are reported by Oldham et al. (Oldham et al., 2008) to be enriched for the same cell types. The pediatric striatum aliceblue (containing 5 NBIA genes) and burlywood (containing *FA2H*) modules also resemble neuronal and oligodendrocytic signatures (Table 3), respectively.

**Fig. 3:**
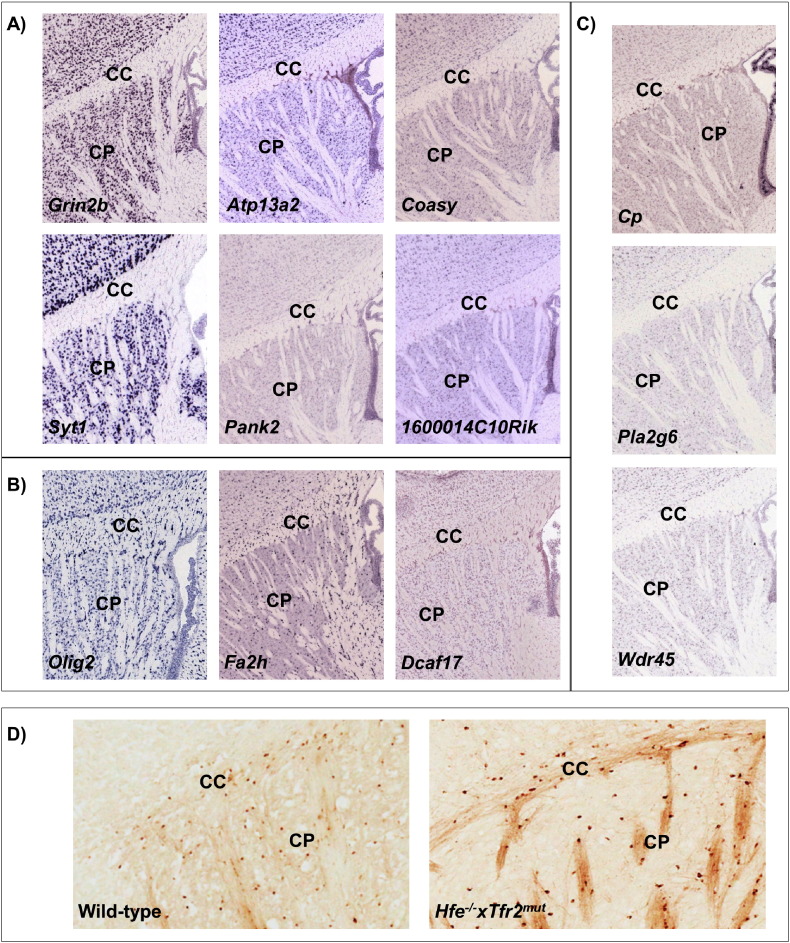
Expression patterns of NBIA genes and neuronal or oligodendrocyte markers (A–C), using data from the Allen Brain Atlas (http://mouse.brain-map.org) (Lein et al., 2007), and patterns of iron accumulation in the brain of wild-type and *Hfe*^*−*/*−*^* xTfr2*^*mut*^ mice (D). A) Genes in the neuronal-enriched module (putamen brown module, *1600014C10Rik* is the mouse ortholog for *C19orf12*); B) Genes in the oligodendrocyte-enriched module (putamen green module, data for *FTL* was not available); C) NBIA genes in other co-expression modules; D) 3,3′-diaminobenzidine-4HCl (DAB)-enhanced Perls' staining shows iron loading in the caudate-putamen and corpus callosum of the *Hfe*^*−*/*−*^* xTfr2*^*mut*^ mice compared with matched wild-type mice. CC — corpus callosum; CP — caudate-putamen.

We further investigated expression patterns of NBIA genes using mouse brain data from the Allen Brain Atlas (http://mouse.brain-map.org) (Lein et al., 2007). NBIA genes in the brown module seem to differentiate gray from white matter and behave similarly to the neuronal markers (Fig. 3), while genes in the green module behave similarly to oligodendrocytic markers. This is also in agreement with the relatively higher expression of green module genes in the white matter.

### 3.4 Basal ganglia NBIA gene networks implicate synapse and lipid metabolism

In line with the cell types described above, in the putamen brown module, which includes *PANK2*, *ATP13A2*, *C19orf12*, and *COASY*, several GO terms related with synaptic transmission, neuron projection development, protein modification by small protein conjugation or removal, modification-dependent protein catabolic process, and synapse are overrepresented. Synaptic vesicle cycle KEGG pathway (KEGG:04721) is also enriched (p = 2.33 × 10^− 4^). We further investigated this module for the presence of genes encoding for well-known synaptic vesicle and synaptic plasma membrane proteins (Morciano et al., 2005). Synaptic vesicle genes *VAMP2*, *SYT1*, and *RAB3A* have high module memberships in the brown module, as do pre- and post-synaptic plasma membrane genes, including *SNAP25*, *ATP1A3*, *DLG2*, *DLG3*, and *DLG4* (all > 75th quantile).

For the green module, which includes *FTL*, *DCAF17*, and *FA2H*, enrichment analysis shows a significant overrepresentation of multiple GO terms, such as ensheathment of neurons, lipid biosynthetic process, membrane organization, myelin sheath, dipeptidase activity, and protein binding. The Sphingolipid metabolism KEGG pathway (KEGG:00600), which is essential for proper myelination, is also overrepresented (p = 1.15 × 10^− 2^). We observed that our NBIA-containing modules comprise at least 21/29 myelination-related genes (as defined by (Hakak et al., 2001)), and the green module alone contains 12, including *MAG*, *MOG*, *PLP1*, and *CNP* (all > 95th quantile).

### 3.5 Basal ganglia NBIA gene networks tightly link to iron metabolism

As the unifying feature in NBIA disorders is focal basal ganglia iron accumulation, we investigated whether iron metabolism-related genes (Rouault, 2013, Constantine et al., 2008) are present in the putamen NBIA-enriched modules (Fig. 2). The brown module contains the iron-responsive element binding protein 2 gene (*IREB2*), a key gene in the regulation of intracellular iron homeostasis. It also includes *SLC25A37*, which encodes for the mitochondrial iron importer mitoferrin 1, as well as *EXOC6* (exocyst complex component 6, which is linked to the transferrin cycle (Lim et al., 2005), hub gene – 91st quantile), *HMOX2* (heme oxygenase), and *PGRMC1* (heme binding protein). The green module, apart from the NBIA *FTL* gene, also includes the ferritin heavy polypeptide 1 (*FTH1*), which are both important for iron sequestration. Additionally, it contains transferrin (*TF*, hub gene – 98th quantile), transferrin receptor (*TFRC*), and solute carrier family 11 member 2 (*SLC11A2* also known as *DMT1*, proton-coupled divalent metal ion transporter) genes, all involved in iron uptake.

An overrepresentation of key iron-related genes is observed in these two gene expression modules (12/36 transcripts, p = 0.007), suggesting that they play a central role in brain iron metabolism. NBIA genes and iron-related genes are interconnected with the same hub genes (Fig. 2), suggesting that perturbations of these networks may underlie the iron metabolism dysregulation seen in NBIA disorders. We observed a significant overlap between genes differentially expressed in post-mortem basal ganglia tissue of NBIA cases compared to matched controls and genes in the green (p < 0.0001) and brown (p = 0.04) modules, suggesting that these networks are indeed disturbed in NBIA brains.

### 3.6 Excessive brain iron loading disturbs basal ganglia NBIA gene networks

To investigate whether the accumulation of iron itself triggers disturbances in NBIA networks, we investigated gene expression changes in an iron overload mouse model (*Hfe*^−/−^ x*Tfr2*^*mut*^) without mutations in NBIA genes. This mouse model shows increased brain iron (Fig. 3) and ferritin levels (> 1.7-fold, p < 0.025) at 12 weeks of age. In both mutant and wild-type mice brains, iron levels increase with age, and iron localizes predominantly in the basal ganglia and the choroid plexus, and overlaps with myelin-rich areas. At age 13 weeks, brain gene expression analysis in male mice reveals differentially expressed genes in the mutants when compared to wild-type (310 upregulated and 451 dowregulated genes, p < 0.05). The mutant mice show downregulation of five NBIA genes (orthologues for *PLA2G6*, *FA2H*, *CP*, *C19orf12*, and *ATP13A2*) (Heidari et al., 2014), and there is excessive overlap between genes in the human NBIA-enriched modules (both green and brown) and genes differentially expressed (mostly downregulated) in the mutant compared to wild-type mice (p < 1.00 × 10^− 4^). Many of these genes are highly interconnected within the NBIA networks (hub genes, > 90th quantile), including for example *CNP* (a myelin-related gene) from the green module, and *ATP6V0A1* (synaptic vesicle-related gene) from the brown module. This suggests that increased brain iron load disturbs the NBIA networks.

Functional enrichment analysis for dysregulated genes in the mutant mice overlapping with green or brown module genes reveals an overrepresentation of GO terms related with the endomembrane system. Phagosome (KEGG:04,145, p = 3.52 × 10^− 2^) and Synaptic vesicle cycle (KEGG:04,721, p = 3.94 × 10^− 2^) pathways are also enriched for genes overlapping with the green and brown modules, respectively. Overall, brain iron overload seems to compromise membrane trafficking by disrupting basal ganglia NBIA gene networks.

### 3.7 Communalities with additional neurological syndromes

We further investigated whether genes in our basal ganglia NBIA networks are associated with additional human neurological disorders with abnormal brain iron content (Dusek et al., 2012, Sipe et al., 2002, Oshiro et al., 2011, Batista-Nascimento et al., 2012). This is the case for genes associated with Mucolipidosis type IV (*MCOLN1*-OMIM# 605248), X-linked sideroblastic anemia with ataxia (*ABCB7*-OMIM#300135), Parkinson's disease (*VPS35*-OMIM#601501, and *SYNJ1*-OMIM#604297), Alzheimer's disease (*ADAM10*-OMIM#602192, and *PLD3*-OMIM#615698), and amyotrophic lateral sclerosis (*UBQLN2*-OMIM#300264, *VCP*-OMIM#601023, *SIGMAR1*-OMIM#601978, and *SOD1*-OMIM#147450). Furthermore, as a proof of principle that these networks provide pools of candidate genes, there are recent reports on NBIA compatible phenotypes associated with mutations in *RAB39B* (OMIM#300774) (Wilson et al., 2014) and *UBQLN2* (OMIM#300264) (Fahed et al., 2014) – two genes belonging to our NBIA-enriched networks.

We also explored Human Phenotype Ontology (HPO) terms associated with the 10 NBIA genes and inferred whether other genes in our networks are associated with the same HPO terms. In the top 15 most significantly enriched terms for NBIAs are core NBIA symptoms, such as Dystonia (HP:0001332, p = 6.79 × 10^− 19^), Dysarthria (HP:0001260, p = 1.23 × 10^− 11^), Cognitive impairment (HP:0100543, p = 1.87 × 10^− 11^), Parkinsonism (HP:0001300, p = 1.01 × 10^− 10^), and Spasticity (HP:0001257, p = 1.96 × 10^− 10^). Crossing our putamen NBIA-enriched modules with genes associated with at least two of those core HPO terms and an OMIM entry, we found genes associated with Parkinson's disease (*SYNJ1*-OMIM#604297), spastic paraplegia (*BSCL2*-OMIM#606158), Lesch-Nyhan syndrome (*HPRT1*-OMIM#308000), and other neurological diseases/syndromes (*ATP6AP2*-OMIM#300556, *L1CAM*-OMIM#308840), among highly interconnected genes (≥ 95th quantile) of the brown module. In the top 5 quantiles of the green module, we found genes associated with spastic paraplegia (*PLP1*-OMIM#300401), Niemann-Pick disease (*NPC1*-OMIM#607623), and Canavan disease (*ASPA*-OMIM#271900).

## 4 Discussion

NBIA disorders share a core set of clinicopathological features, including neurodegeneration, but not much is known about the originating cell type. According to our data and in line with NBIA histopathological features, multiple cell types are likely to be involved. Neuronally-derived eosinophilic spheroid bodies, thought to represent degenerating neurons and accumulation of protein and lipid storage material as well as damaged organelles (e.g. mitochondria and vesicles), are a pathologic hallmark of several NBIA disorders, including those caused by mutations in *PANK2* and *C19orf12* (Kruer, 2013) — genes that belong to a co-expression module that reflects neuronal signatures. Myelin loss has been associated with *FTL* mutations (Kruer, 2013) and *FA2H* deficiency (Zoller et al., 2008, Potter et al., 2011) — genes of a module that reflects oligodendrocytic signatures and is associated with myelination. Indeed, factors involved in myelination, namely *FA2H*, are gaining relevance in brain disease (e.g. autism spectrum disorders Scheid et al., 2013). Enlarged and distorted iron-overloaded astrocytes (Kruer, 2013) are a core pathological feature in NBIA patients with *CP* mutations, and this gene is in an astrocytic-like module. This multitude of cellular origins suggests that neuronal death in NBIA disorders can result from direct insults to neurons or as secondary events caused by the loss of support normally provided by astrocytes and/or oligodendrocytes.

Dysfunction of membrane trafficking is a hallmark of many neurological and psychiatric diseases (Wang et al., 2013, Kitagishi et al., 2015), with a decreased degradation capacity of pre- and post-synaptic trafficking compartments leading to the accumulation of dysfunctional intracellular machineries (Wang et al., 2013). Our data shows the involvement of the synapse and the endomembrane system in the NBIA networks. It is possible that the characteristic NBIA spheroids are a reflection of these events, with consequent neurodegeneration due to the inherent toxicity of the cargo overload or a toxic cellular response to such overload (Wang et al., 2013). NBIAs share the variable accumulation of α-synuclein-positive Lewy bodies and/or tau pathology and brain iron deposition with common neurodegenerative diseases (e.g. Parkinson's and Alzheimer's diseases (Kruer, 2013)). A better understanding of the synaptic pathology in NBIAs raises the hope for the development of therapeutic strategies that will improve synaptic maintenance, which is essential for neuronal health, and help to therapeutically tackle NBIAs and more common neurological, psychiatric and neurodevelopmental diseases sharing underlying pathology.

Iron is essential for normal neurological function, as it is required for the production of high levels of ATP needed to maintain membrane ionic gradients, synaptic transmission, axonal transport, neurotransmitter synthesis, myelination, etc (Piñero and Connor, 2000). The brain tends to accumulate iron with age, and the globus palllidus, red nucleus, substantia nigra, and caudate-putamen have higher concentrations of iron throughout life (Piñero and Connor, 2000). Pronounced and premature iron accumulation in the basal ganglia is a hallmark of NBIA disorders, which probably involves loss of concordant regulation between iron uptake, storage and transport within the brain.

Only two NBIA genes (*FTL* and *CP*) have been so far directly implicated in iron metabolism. While mutations in *FTL* disrupt the structure of ferritin and modify its capacity to incorporate iron (Luscieti et al., 2010), mutations in *CP* lead to defective export of iron from cells (di Patti et al., 2009). We showed important connections of iron-related genes within the basal ganglia NBIA networks, indicating a broader involvement of NBIA genes in iron-related processes. Deficiency of the *IREB2*, a gene present in our networks and a key regulator of intracellular iron homeostasis (Rouault, 2013), is enough to cause progressive neurodegeneration with prominent caudate-putamen iron accumulation in mice (LaVaute et al., 2001). Genes involved in iron uptake (*TF*, *TFRC*, and *DMT1*) and storage (*FTL* and *FTH1*) are present as well. Therefore, disruptions in these networks (e.g. by mutations in NBIA genes) likely dysregulate iron-related processes.

We have also shown that brain iron overload can be associated with dysregulated expression of genes present in the NBIA networks, including downregulation of several NBIA genes, even in the absence of mutations in NBIA genes. Altogether, this raises the hypothesis that disturbances in NBIA gene networks contribute to dysregulation of iron metabolism and, in turn, progressive increase in brain iron levels (e.g. with aging) aggravates the disruption of these gene networks. According to this hypothesis, iron accumulation is not mandatory for the onset of the symptoms, but it seems essential in determining the fate of disease progression. This is consistent with the fact that not all patients with mutations in NBIA genes show significant brain iron overload in early stages of the disease (Johnstone et al., 2013, Potter et al., 2011). Whether this finding merely reflects the incapacity of MRI methods to detect subtle iron level changes remains debatable. A recent report with promising results on the stabilization of the disease upon treatment with an iron-chelating agent (Cossu et al., 2014) lends further support to that hypothesis.

In conclusion, our human brain gene co-expression network analysis suggests that multiple cell types act in the origin of the clinically heterogeneous group of NBIA disorders, and reveals strong links with iron-related processes. Overall, our results show convergent pathways connecting groups of NBIA genes and other neurological diseases genes, providing possible points for therapeutic intervention. Given the enrichment of these networks for genes associated with NBIA and overlapping phenotypes, they provide reservoirs of candidate genes useful for prioritizing genetic variants and boosting gene discovery in ongoing collaborative sequencing initiatives.

## Conflicts of interest

The authors declare no conflict of interest.

## Figures and Tables

**Table 1:** Neurodegeneration with brain iron accumulation disorders.

Disease	Gene	OMIM	Inheritance	Disease onset	MRI changes			Neuropathologic findings in humans
Iron deposition	White Matter changes	Other findings
Aceruloplasminemia (ACP)	*CP*	117700	AR	Adulthood	Globus pallidus, putamen, caudate, thalamus, red and dentate nuclei	Moderate, severe	Mild cerebellar atrophy	Globular deposits (grumose or foamy spheroid bodies) in astrocytes > neurons
Neuroferritinopathy (FTL)	*FTL*	134790	AD	Adolescence to adulthood	Globus pallidus, putamen, caudate, red and dentate nuclei, thalamus	Mild, moderate	Cystic cavitation/necrosis in basal ganglia, mild cerebral, cerebellar atrophy	Distorted, iron-laden nuclei
Pantothenate kinase-associated neurodegeneration (PKAN)	*PANK2*	606157	AR	Typically childhood	Globus pallidus, substantia nigra (mild)	None reported	“Eye-of-the-tiger” sign	neurofibrillary tangles, spheroids
COASY protein-associated neurodegeneration (CoPAN)	*COASY*	609855	AR	Childhood	Globus pallidus^a^, substantia nigra^a^	NA	“Eye-of-the-tiger” sign	NA
PLA2G6-associated neurodegeneration (PLAN)	*PLA2G6*	603604	AR	Childhood to adulthood	Globus pallidus, substantia nigra^a^	Mild	Moderate cerebral and cerebellar atrophy	Lewy bodies, neurofibrillary tangles, spheroids
Mitochondrial membrane protein-associated neurodegeneration (MPAN)	*C19orf12*	614297	AR	Typically childhood	Globus pallidus, substantia nigra	NA	Myelin deficit on pyramidal tracts and optic nerve^a^	Lewy bodies, spheroids, hyperphosphorylated tau inclusions
Fatty acid hydroxylase-associated neurodegeneration (FAHN)	*FA2H*	611026	AR	Typically childhood	Globus pallidus, substantia nigra^a^	Moderate	Pontocerebellar atrophy, thin corpus callosum	NA
Kufor-Rakeb syndrome (KRS)	*ATP13A2*	610513	AR	Adolescence	Globus pallidus^a^, putamen^a^, caudate^a^	NA	Severe cerebral, cerebellar, brain stem atrophy	NA
Beta-propeller protein-associated neurodegeneration (BPAN)	*WDR45*	300526	XLD	Adolescence to adulthood	Globus pallidus^a^, substantia nigra^a^	Mild	Mild cerebellar atrophy	NA
Woodhouse-Sakati syndrome (WSS)	*DCAF17* (*C2orf37*)	612515	AR	Adolescence to adulthood	Globus pallidus^a^	Moderate to severe, confluent	NA	NA

NA — no information available.

a Inconsistent findings or very small number of cases.

**Table 2:** NBIA genes/transcripts assigned to basal ganglia gene co-expression modules.

NBIA gene	Affy transcript ID	Putamen		Substantia Nigra	
Module (size)	Quantile	Module (size)	Quantile
***PANK2***	3874533	Brown (1696)	**56**	Brown (1696)	**88**

***COASY***	3721851	25	**54**
***ATP13A2***	2398736	**54**	Yellow (1902)	**90**
***C19orf12***	3857808	**57**	13
	3857811	30	**55**
***FTL***	3838094	Green (950)	24	Purple (401)	**64**

***DCAF17***	2515183		37	Red (1099)	**63**
***FA2H***	3699133		**84**	Green (1483)	**92**

***CP***	2700244	Greenyellow (171)	35	Red (900)	42

***PLA2G6***	3960388	Turquoise (6888)	19	Green (1437)	37
***WDR45***	4007774	**80**	Turquoise (4109)	**87**

Size — number of genes in the module; Quantile — quantile for module membership.

Module memberships above the median are highlighted in bold.

**Table 3:** Basal ganglia NBIA-containing modules are enriched for cell-specific markers.

Network	NBIA-containing modules	Oligodendrocytes^a^	Astrocytes^a^	Neurons^a^
Adult putamen	Brown	176/1537	128/1878	**375/1445** **(p** **=** **4.70 × 10**^**− 51**^**)**
Green	**303/1537** **(p** **=** **5.94 × 10**^**− 60**^**)**	103/1878	45/1445
Greenyellow	10/1537	**14/1878** **(p** **=** **8.37 × 10**^**− 4**^**)**	4/1445
Turquoise	289/1537	**447/1878** **(p** **=** **0.002)**	315/1445

Pediatric striatum	Aliceblue	263/1384	362/1728	**238/1308** **(p** **=** **1.53 × 10**^**− 4**^**)**
Bisque3	205/1384	234/1728	182/1308
Burlywood	**26/1384** **(p** **=** **1.21 × 10**^**− 4**^**)**	9/1728	9/1308
Darkolivegreen1	243/1384	272/1728	**365/1308** **(p** **=** **2.25 × 10**^**− 17**^**)**
Green4	17/1384	9/1728	12/1308
Lightpink3	22/1384	25/1728	4/1308

Enrichment analysis for Oligodendrocytes, Astrocytes and Neurons-enriched genes in NBIA-containing modules. Fractions represent the number of overlapping genes relative to the maximum possible overlapping genes. Significance was evaluated using Fisher's exact test (only p-values < 0.05 are shown).

Statistically significant enrichments for specific cell types are highlighted in bold.

a Genes enriched in major cell types as defined by Cahoy et al. (2008).
